# Phospholipase D1 inhibition sensitizes glioblastoma to temozolomide and suppresses its tumorigenicity

**DOI:** 10.1002/path.5519

**Published:** 2020-09-10

**Authors:** Dong Woo Kang, Won Chan Hwang, Yu Na Noh, Kang Seo Park, Do Sik Min

**Affiliations:** ^1^ Department of Molecular Biology, College of Natural Science Pusan National University Busan Republic of Korea; ^2^ College of Pharmacy Yonsei University Incheon Republic of Korea; ^3^ Asan Institute for Life Science Asan Medical Center Seoul Republic of Korea; ^4^ Department of Oncology, Asan Medical Center University of Ulsan College of Medicine Seoul Republic of Korea

**Keywords:** phospholipase D1, glioblastoma, temozolomide, miRNA‐320a/‐4496, temozolomide resistance factor

## Abstract

Resistance of glioblastoma to the chemotherapeutic compound temozolomide is associated with the presence of glioblastoma stem cells in glioblastoma and is a key obstacle for the poor prognosis of glioblastoma. Here, we show that phospholipase D1 is elevated in CD44^High^ glioblastoma stem cells and in glioblastoma, especially recurring glioblastoma. Phospholipase D1 elevation positively correlated with the level of CD44 and poor prognosis in glioblastoma patients. Temozolomide significantly upregulated the expression of phospholipase D1 in the low and moderate CD44 populations of glioblastoma stem cells, but not in the CD44^High^ population in which phospholipase D1 is highly expressed. Phospholipase D1 conferred resistance to temozolomide in CD44^High^ glioblastoma stem cells and increased their self‐renewal capacity and maintenance. Phospholipase D1 expression significantly correlated with levels of temozolomide resistance factors, which were suppressed by microRNA‐320a and ‐4496 induced by phospholipase D1 inhibition. Genetic and pharmacological targeting of phospholipase D1 attenuated glioblastoma stem cell‐derived intracranial tumors of glioblastoma using the microRNAs, and improved survival. Treatment solely with temozolomide produced no benefits on the glioblastoma, whereas in combination, phospholipase D1 inhibition sensitized glioblastoma stem cells to temozolomide and reduced glioblastoma tumorigenesis. Together, these findings indicate that phospholipase D1 inhibition might overcome resistance to temozolomide and represents a potential treatment strategy for glioblastoma. © 2020 The Authors. *The Journal of Pathology* published by John Wiley & Sons, Ltd. on behalf of The Pathological Society of Great Britain and Ireland.

## Introduction

Phospholipase D (PLD) has been implicated in various biological processes, including cancer [1, 2, 3]. PLD catalyzes hydrolysis of phosphatidylcholine (PC) to generate phosphatidic acid (PA), which activates a signaling cascade for cell growth and survival. Two isoforms of PC‐specific PLD, PLD1 and PLD2, have been identified [2]. PLD is highly upregulated in diverse cancers, and its expression is positively correlated with tumor malignancy, invasiveness, and survival [1, 2, 4, 5, 6]. Glioblastoma (GBM) is a highly lethal brain tumor comprising tumor‐propagating glioma stem cells (GSCs), which promote therapeutic resistance and tumor recurrence with poor prognosis. The alkylating drug temozolomide (TMZ) is routinely used [7]. However, drug resistance is a major hurdle. PLD has pivotal roles in tumorigenesis and cancer progression, and radio‐/chemo‐resistance [3, 4, 5, 8]. PLD1 regulates the migration, proliferation, and viability of GBM cells [9, 10, 11, 12, 13]. However, the role of PLD1 in TMZ chemoresistance and management of GBM *in vivo* remain unknown. Targeting PLD1 attenuates intestinal tumorigenesis by blocking phosphoinositide 3‐kinase/Akt and Wnt/β‐catenin signaling pathways [4], which are associated with glioma development and poor prognosis of GBM patients [14]. Thus, PLD and its associated signaling pathways may be clinically important therapeutic targets. How PLD contributes to TMZ resistance in GSCs is unclear. TMZ induces DNA methylation of guanine at the O^6^ position, which leads to double‐strand breakage, cell‐cycle arrest, and apoptosis. *O* ^6^‐metG is repaired by *O* ^6^‐methylguanine DNA methyltransferase (MGMT). Although elevated MGMT expression mainly contributes to TMZ resistance, MGMT alone does not fully account for the TMZ chemoresistance of GBM [15]. Several other genes are also reportedly involved. We searched for other mechanisms that may contribute to TMZ resistance by investigating the contribution of PLD1 in TMZ resistance and exploring the therapeutic implications for targeting PLD1 in TMZ‐resistant GSCs. Targeting PLD1 alone or in combination with TMZ attenuated GBM tumorigenicity through microRNA (miR)‐320a and miR‐4496‐mediated downregulation of TMZ resistance factors, including MGMT.

## Materials and methods

### Cell lines and culture

U251‐MG (#09063001) human GBM cells were obtained from the European Collection of Authenticated Cell Cultures. GL26 and GL261 murine GBM cells were provided by Professors H Phillip Koeffler (University of California at Los Angeles) and John R Ohlfest (University of Minnesota). To establish TMZ resistance, U251‐MG cells were cultured in the presence of a low dose of TMZ for 6 months. TMZ‐resistant cells were designated U251‐TMZ‐R. The IC_50_ values for growth inhibition of U‐251MG and U251‐TMZ‐R after exposure to TMZ were 21.6 and 271.3 μm, respectively. Human GSC (GSC_X01, GSC_X02, GSC_X03, GSC83, GSC84, GSC131, GSC528) cells obtained from Dr DH Nam (Samsung Medical Center) were maintained in sphere culture conditions. For serial passage, the cell spheres were dissociated to single cells using Accutase (#A1110501; Thermo Fisher Scientific, Waltham, MA, USA) every 5–7 days, and further incubated under previously described culture conditions [16, 17, 18, 19, 20].

### Isolation of different cancer stem cell (CSC) subpopulations

CD44^+^ cells were sorted using magnetic‐activated cell sorting. Dissociated tumor cells were incubated with CD44 MicroBeads for 30 min at 4 °C, followed by cleavage of the MicroBeads. To further enrich for epithelial cells, CD31/CD45 depletion was performed using the MACS‐based protocol with MicroBeads conjugated with monoclonal antibody to CD44 (#130‐095‐194), CD45 (#130‐045‐801), and CD31 (#130‐097‐418) (all from Miltenyi Biotec, Bergisch Gladbach, Germany). Purity of the isolated subpopulations regularly exceeded 90%. Fluorescence‐activated cell sorting (FACS) analyses and sorting were paired with the matched isotype control. The antibodies used included CD45‐fluorescein isothiocyanate (FITC, #555482; BD Biosciences, San Jose, CA, USA), CD31‐BV‐421 (#744462; BD Biosciences), and CD44‐allophycocyanin (APC, #559942; BD Biosciences).

### Isolation of neural progenitor cells (NPCs) from brains of mice

Murine NPCs (mNPCs) were isolated from the brains of mice and characterized as previously described [21]. Isolated mNPCs were grown in suspension culture by three passages in supplemented neurobasal stem cell medium.

### FACS and flow cytometry

APC‐conjugated anti‐CD44 (#103011, 1:200; Biolegend, San Diego, CA, USA), phycoerythrin (PE)‐conjugated anti‐glial fibrillary acidic protein (*GFAP*; #FCMAB257P, 1:40; Millipore, Burlington, MA, USA), PE‐conjugated anti‐microtubule‐associated protein 2 (*MAP2*; #FCMAB318PE, 20 μl per test; Millipore), and control IgG (#555749, 1:40; BD Biosciences) antibodies were used for FACS. To obtain CD44^High^ or CD44^Low^ cells, patient‐derived primary tumor cells or transplanted murine primary tumor cells stained with 1:200 dilutions of APC‐conjugated anti‐CD44 antibody were subjected to FACS. For cell cycle analyses, cells were incubated with 20 μm BrdU (#B5002; Sigma‐Aldrich, St Louis, MO, USA) for 3 days, followed by incubation with PE‐conjugated anti‐BrdU antibody (#339811, 1:40; Biolegend). Apoptotic cell death was measured using an APC‐ (#ab236215; Abcam, Cambridge, UK) or FITC‐conjugated (#ab14085; Abcam) Annexin V Apoptosis Detection Kit I.

### Intracranial tumor formation *in vivo*

Animal studies were approved by the Institutional Animal Care Committee of Pusan National University (Permit No: 2013‐0403). Intracranial transplantation of GSCs to establish GBM xenografts was performed [22]. Briefly, patient‐derived CD44^High^, CD44^Negative^ GSCs, and GL26‐transplanted CD44^High^ cells were transduced with short hairpin control (sh‐CTRL), sh‐*PLD1*, anti‐miR‐4496/320a, or control vector through lentiviral infection twice, with a 24 h interval. At 24 h after the second transduction, viable cells were injected intracranially using a stereotactic device at a depth of 3 mm into the right forebrains of 9‐ to 10‐week‐old immunocompromised male NOD‐SCID mice (6 × 10^4^ cells per mouse) or male syngeneic C57BL/6 mice (2 × 10^4^ cells per mouse). The mice were anesthetized with tribromoethanol (250 mg/kg intraperitoneally, T48402; Sigma‐Aldrich). All mice bearing the indicated cells were injected intraperitoneally with TMZ (20 mg/kg) three times weekly or with PLD1 inhibitor (VU0155069, 10 mg/kg) five times weekly. In survival experiments, animals were maintained until manifestation of neurological signs or for 120 days.

### Statistical analyses

Data were analyzed using Student's *t*‐test. Correlation coefficients were calculated using Spearman's rho (*r*). Glioma survival probability (time from brain resection to death or date of last follow‐up) was determined using Kaplan–Meier analysis, and differences were evaluated using the log‐rank test. Immunohistochemical staining results were analyzed by the chi‐squared test. Statistical analyses were performed using Origin 8.0 software (OriginLab, Northampton, MA, USA) and GraphPad Prism 5.0 software (GraphPad Inc, San Diego, CA, USA).

Details of the following methods are presented in supplementary material, Supplementary materials and methods: PLD activity assay; Reagents and radiation exposure; Preparation of plasmids, shRNAs, and miRNA; Viral production and infection; Transient transfection and reporter gene assay; Reverse transcription‐quantitative PCR; Quantification of mature miRNA; 3′‐UTR reporter constructs; Cell viability assessment using trypan blue exclusion; Differentiation assay; *In vitro* limiting dilution assay (LDA); Immunoblotting; Immunohistochemistry (IHC); and Expression data from the TCGA glioblastoma multiforme dataset.

## Results

### Upregulation of PLD1 is associated with poor prognosis in GBM

**Figure 1:**
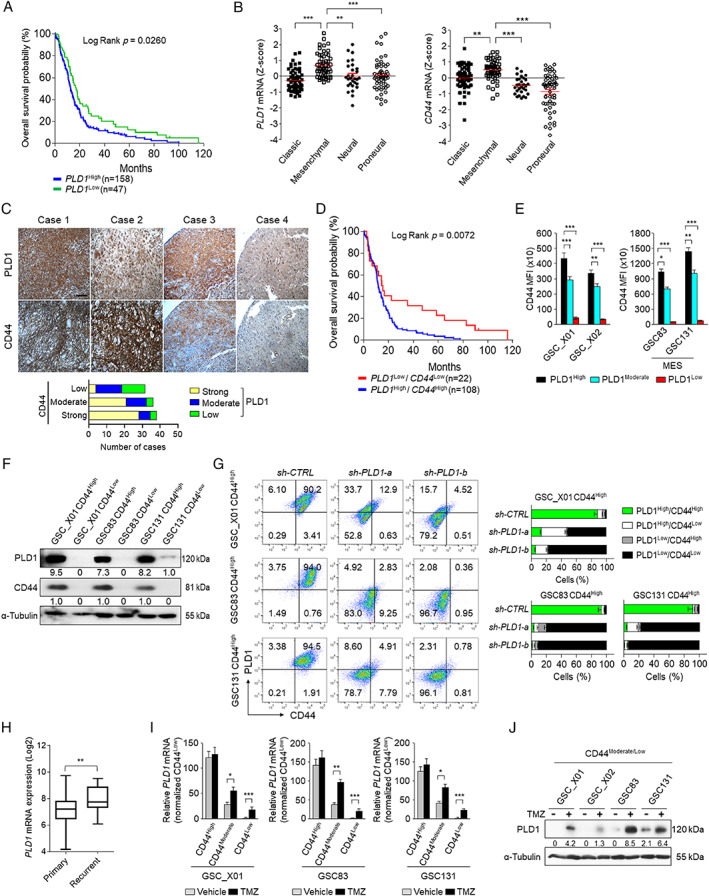
PLD1 is upregulated in GBM tissues, CD44^High^ GSCs, and TMZ‐treated CD44^Moderate^/^Low^ GSCs, and correlates with poor prognosis in GBM patients. (A) Overall survival probability associated with PLD1 expression in TCGA GBM datasets. (B) Expression of PLD1 and CD44 in TCGA datasets of the four subtypes of GBM. (C) The upper panels show representative images of four tumors (bar = 50 μm). The lower panel contains graphs summarizing IHC assays using anti‐PLD1 and anti‐CD44 antibodies in 103 human high‐grade glioma specimens. (D) Overall survival probability in relation to levels of *PLD1* and *CD44* in the TCGA GBM dataset. Survival probability was determined using Kaplan–Meier analysis. (E) The levels of PLD1 in the indicated populations of CD44 of GSCs sorted by flow cytometry. (F) The protein levels of PLD1 and CD44 in the high and low CD44 populations of GSCs sorted by flow cytometry. (G) Effect of PLD1 depletion on the CD44 population in the indicated GSCs analyzed by flow cytometry. (H) Expression of *PLD1* mRNA in the TCGA GBM dataset with primary tumors (*n* = 153) and recurrent tumors (*n* = 13) of GBM patients. (I) Effect of TMZ (50 μm, 48 h) on the expression of *PLD1* mRNA in the CD44 populations from various GSCs. (J) Effect of TMZ on the protein levels of PLD1 in the CD44^Moderate/Low^ population of GSCs. Results are representative of at least three independent experiments and are presented as the mean ± SEM (D, H–J). *p < 0.05; **p < 0.01; ***p < 0.001, by Student's *t*‐test.

We investigated whether PLD1 expression is altered in GBM tissues using immunohistochemistry (IHC). Immunoreactivity for PLD1 was quantified (strong, moderate, and weak staining intensity). PLD1 expression was highly elevated in human GBM tissues, relative to normal regions of the brain (supplementary material, Figure S1). PLD1 expression was correlated with poor prognosis of GBM patients in cohorts based on The Cancer Genome Atlas (TCGA) GBM expression profile database (Cancer Genome Atlas Research Network, 2008; https://tcga-data.nci.nih.gov/tcga/tcgaDownload.jsp) (Figure 1A). GBM comprises four clinically relevant subtypes: classic, mesenchymal (MES), neural, and proneural (PN) [23, 24]. The MES subtype is more aggressive and radioresistant than PN‐GBM [17]. PLD1 and CD44 expression was significantly elevated in the MES subtype, compared with the other subtypes (Figure 1B). CD44 is a marker of the MES subtype and worse prognosis, and is a predictor of radioresistance in GBM [25]. We further evaluated the pathological correlation between PLD1 and CD44, using 103 high‐grade brain tissue microarrays (TMAs). The representative IHC images in Figure 1C show four different cases based on immunoreactivities of PLD1 and CD44. The intensity of immunoreactivity revealed a correlation between the levels of PLD1 with those of CD44 (Figure 1C). The prognosis of GBM patients with high levels of PLD1 and CD44 was poor, compared with cohorts with low levels of PLD1 and CD44 in the TCGA (Cancer Genome Atlas Research Network, 2008, Figure 1D). Patient‐derived GSC_X01 or GSC_X02 [16, 18, 20], and MES‐GSCs (GSC83, GSC131) [19], which display high PLD1 expression, displayed a higher CD44 population (Figure 1E). Additionally, PLD1 was dramatically elevated in CD44^High^ GSCs compared with CD44^Low^ GSCs (Figure 1F). CD44^High^ MES‐GSCs displayed increased PLD activity and sphere formation relative to CD44^Low^ GSCs (supplementary material, Figure S2A,B). PLD1 depletion significantly reduced CD44^High^ and increased CD44^Low^ populations of various GSCs (Figure 1G). Recurrent GBM is characterized by radio/chemotherapy resistance and poor clinical prognosis. Mining the GBM data portal of the TCGA revealed that PLD1 expression was significantly higher in recurrent GBM tumors than in primary GBM tumors (Figure 1H). Interestingly, TMZ significantly upregulated the expression of PLD1 in CD44^Low^ and CD44^Moderate^ GSCs, but not in CD44^High^ cells in which PLD1 was highly expressed (Figure 1I,J). These results indicate that upregulation of PLD1 is associated with poor prognosis in GBM.

### PLD1 confers resistance to TMZ, self‐renewal capacity, and maintenance of GSCs

**Figure 2:**
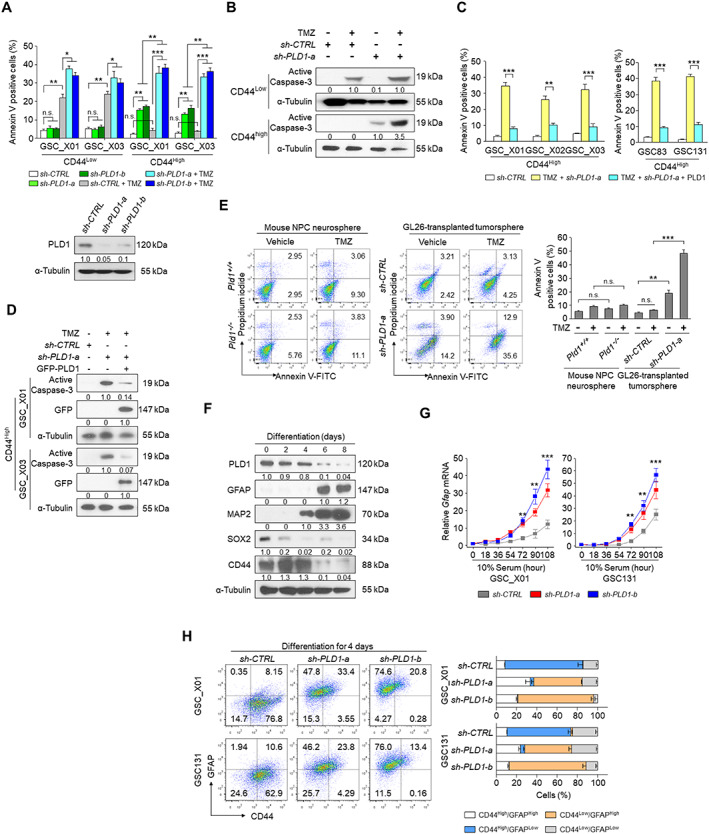
PLD1 contributes to TMZ resistance in the CD44^High^ population of GSCs and maintenance of GSCs. (A) Following transduction with control shRNA (sh‐CTRL) and PLD1‐shRNAs (sh‐PLD1‐a, ‐b), GSCs were exposed to TMZ (100 μm) for 72 h. Apoptosis was analyzed using an Annexin V‐FITC Apoptosis detection kit. The level of PLD1 protein in PLD1‐depleted cells is shown. (B) Effect of TMZ on the level of active caspase‐3 in the GSC_X01 cells expressing sh‐CTRL and sh‐PLD1‐a. (C) Effect of overexpression of PLD1 on the apoptosis induced by TMZ and PLD1 depletion. (D) Effect of PLD1 overexpression on the level of active caspase‐3 induced by TMZ and PLD1 depletion. (E) Effect of TMZ and/or PLD1 depletion on the apoptosis in neurospheres of NPCs from *Pld1* ^+/+^ and *Pld1* ^−/−^ mice, and GL26‐transplanted tumorspheres. (F) Western blotting analysis of the indicated proteins during differentiation of GSC‐X01. (G) Effect of PLD1 depletion on GFAP expression during differentiation of GSCs, as analyzed by qPCR. (H) Effect of PLD1 depletion on the population of CD44 and GFAP during differentiation of the indicated GSCs. Results are representative of at least three independent experiments and are presented as the mean ± SEM (A, C, G). *p < 0.05; **p < 0.01; ***p < 0.001, by Student's *t*‐test; n.s., not significant.

Since upregulation of PLD1 is associated with chemoresistance in various cancer cells [1, 2, 3], we examined whether PLD1 affects resistance to TMZ chemotherapy in GSCs. TMZ induced apoptosis in CD44^Low^ GSCs. PLD1 depletion had no effect on apoptosis, probably because of the low expression of PLD1 in CD44^Low^ cells (Figure 2A). However, PLD1 depletion further increased the TMZ‐induced apoptosis in CD44^Low^ cells (Figure 2A), perhaps reflecting TMZ‐induced PLD1 upregulation (Figure 2A). Conversely, PLD1 depletion, but not TMZ treatment, enhanced the apoptosis of CD44^High^ cells (Figure 2A). The effects of PLD1 depletion and TMZ on caspase‐3 activation in CD44^High^ GSCs (Figure 2B) were also comparable to those shown in Figure 2A. Moreover, overexpression of PLD1 protected against apoptosis induced by TMZ and PLD1 depletion (Figure 2C,D). TMZ in combination with ionizing radiation (IR) is the current standard treatment for GBM. In the present study, TMZ or IR alone did not affect the viability of CD44^High^ GSCs, but their combined treatment significantly decreased the viability of the GSCs (supplementary material, Figure S3A). PLD1 depletion in combination with TMZ or IR further reduced the viability of GSCs compared with the TMZ and IR combined treatment (supplementary material, Figure S3A). Since GSC‐directed therapies could be toxic to normal stem cells due to their shared molecular characteristics, we assessed the therapeutic margin for PLD1‐directed treatment strategies using NPCs. TMZ had no effect on the apoptosis of NPCs derived from PLD1 wild‐type and null mice (Figure 2E). PLD1 depletion, but not treatment with TMZ, significantly increased apoptosis in the GSCs derived from tumors transplanted with GL26 mouse GBM (Figure 2E). Co‐treatment involving TMZ and PLD1 depletion further promoted the apoptosis of GSCs, relative to PLD1 depletion alone. PLD1 and TMZ had no effect on the sphere formation of NPCs derived from mice. PLD1 depletion, but not TMZ treatment, significantly inhibited the tumorsphere formation in GSCs derived from tumors transplanted with GL26 or GL261 cells (supplementary material, Figure S3B). The combined treatment with PLD1 depletion and TMZ further suppressed tumorsphere formation of GSCs relative to either treatment alone. To investigate whether PLD1 could affect the capacity for serial neurosphere formation, serial dilutions of the CD44^High^ GSCs were replated for secondary sphere‐forming assays. PLD1 depletion significantly reduced the mean sphere‐forming capacity of GSCs by an average of 200‐fold based on *in vitro* limiting dilution assay (LDA) (supplementary material, Figure S3C), suggesting that PLD1 regulates the self‐renewal capacity of GSCs. We further investigated the functional link between PLD1 and maintenance of GSCs during GSC differentiation. This differentiation was associated with the downregulation of PLD1 and stemness markers (SOX2 and CD44), and the upregulation of GFAP, a marker of astrocyte differentiation, and MAP2, a marker of neuronal differentiation (Figure 2F), suggesting that PLD1 might be associated with GSC maintenance. PLD1 depletion resulted in increased expression of *GFAP*, but not *MAP2*, during differentiation of GSCs (Figure 2G and supplementary material, Figure S4A), indicating that PLD1 might regulate astrocytic differentiation of GSCs. During this differentiation, PLD1 depletion reduced the CD44^High^/GFAP^Low^ population, but increased the CD44^Low^/GFAP^High^ population (Figure 2H). However, PLD1 depletion had a marginal effect on the CD44^Low^/MAP2^High^ population (supplementary material, Figure S4B). These findings indicate that PLD1 confers resistance to TMZ in the CD44^High^ population of GSCs, self‐renewal capacity, and maintenance of GSCs.

### Combination of PLD1 depletion and TMZ sensitizes GSC‐derived intracranial tumors to TMZ

**Figure 3:**
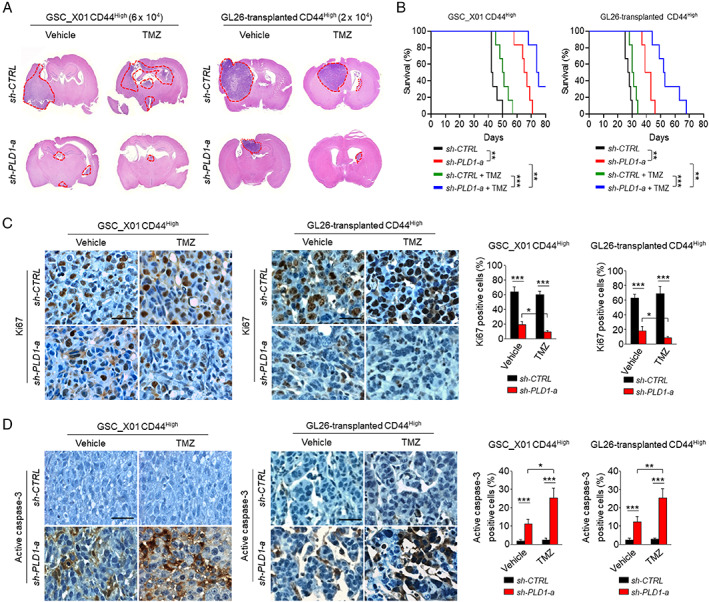
Combination of PLD1 depletion and TMZ sensitizes GSC‐derived intracranial tumors to TMZ. (A, B) Mice intracranially implanted with sh‐PLD1‐a‐ and sh‐CTRL‐expressing GSCs were treated with TMZ three times a week (*n* = 6 per group). Representative images of hematoxylin and eosin‐stained sections of mice brains harvested on day 35 and day 24 after transplantation of the indicated groups, respectively. The areas of red dotted lines indicate the brain tumor sizes in the indicated group (A). Survival of mice was evaluated using the Kaplan–Meier model with two‐sided log‐rank test (B). **p < 0.01; ***p < 0.001. (C) Representative images of IHC (left) and quantification of Ki67‐positive cell population in tumors of mice bearing the indicated GSCs (right). Bar = 100 μm. (D) Representative IHC (left) and quantification of active caspase‐3‐positive cells in tumors of mice bearing the indicated GSCs (right). Bar = 100 μm. Results are representative of at least three independent experiments and are presented as the mean ± SEM (C, D). *p < 0.05; **p < 0.01; ***p < 0.001, by Student's *t*‐test.

We further investigated the effects of PLD1 depletion and/or TMZ on the tumor‐propagating capacity of GSCs. The CD44^High^ population of the GSC_X01 or CD44^High^ population isolated from mouse GL26‐transplanted tumors was transduced with sh‐*PLD1*, followed by culture under conditions that allowed sphere formation. The GSCs were subsequently transplanted into the brains of immunocompromised NOD‐SCID mice and syngeneic C57BL/6 mice. Mice intracranially implanted with the CD44^High^ GSCs transduced with sh‐*PLD1* displayed markedly reduced tumor formation (Figure 3A) and significantly increased survival, relative to those bearing GSCs expressing control shRNA (Figure 3B). Treatment with TMZ had marginal effects on tumor formation and survival (Figure 3A,B). However, PLD1 depletion and TMZ treatment remarkably suppressed intracranial tumor formation and increased survival. We further examined the expression of caspase‐3 and Ki‐67 in tumors using IHC. GBM tumors derived from PLD1‐depleted GSCs showed lower proportions of Ki67‐positive proliferating cells (Figure 3C). Although TMZ alone had a marginal effect on proliferation, administration of TMZ in mice bearing PLD1‐depleted GSCs further decreased the number of Ki67‐positive cells (Figure 3C). TMZ had a marginal effect on the apoptosis of GBM tumors, but PLD1 depletion significantly increased the portion of active caspase‐3‐positive cells (Figure 3D). The combination treatment further promoted the apoptosis of GBM tumors (Figure 3D). These results indicate that PLD1 depletion sensitizes the effect of TMZ on GSC‐derived intracranial tumors of GBM.

### PLD1 depletion sensitizes resistance against TMZ in GSCs via miR‐320a‐ and miR‐4496‐mediated downregulation of TMZ resistance factors

**Figure 4:**
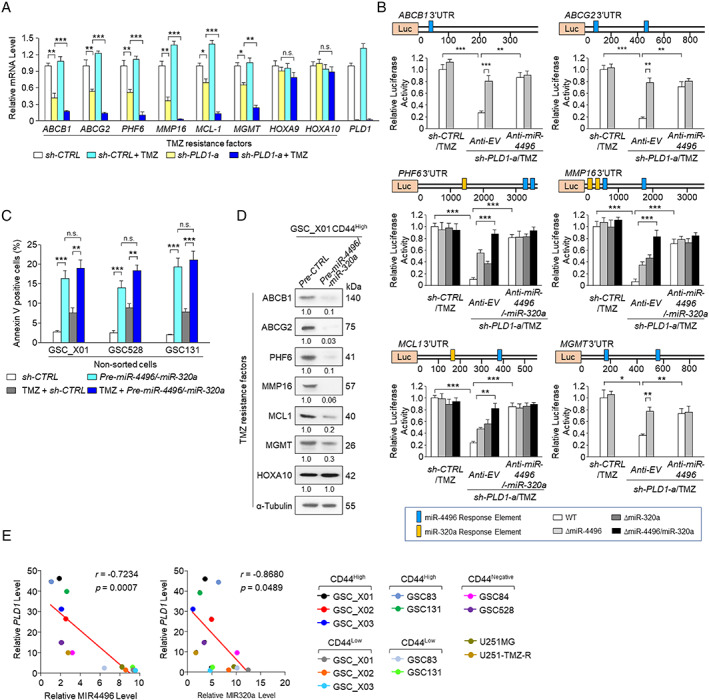
PLD1 depletion sensitizes resistance against TMZ in GSCs via miR‐320a and **‐**4496‐mediated downregulation of TMZ resistance factors. (A) qPCR analysis of the indicated TMZ resistance factors and PLD1 in GSC_X01 transduced with sh‐PLD1‐a and treated with or without TMZ (100 μm) for 72 h. (B) Schematic representation of luciferase constructs containing wild‐type (WT) and mutant binding sites of miR‐320a and ‐4496 in 3′‐UTR reporters of the TMZ resistance genes. Luciferase assays of 3′‐UTR reporters of the genes under the indicated condition. (C) Effect of pre‐miR‐4496/‐miR‐320a on the apoptosis in the indicated GSCs that were untreated or treated with TMZ (100 μm). (D) Effect of pre‐miR‐320a/‐4496 on expression levels of the TMZ resistance factors in GSCs. (E) Inverse correlation of the *PLD1* mRNA level with miR‐4496 or miR‐320a expression in the indicated cells. Spearman's correlation coefficient (*r*) is provided with its statistical significance. The red line represents the best‐fit curve. Results are representative of at least three independent experiments and are presented as the mean ± SEM (A, C, D). *p < 0.05; **p < 0.01; ***p < 0.001, by Student's *t*‐test; n.s., not significant.

Since several genes are reportedly involved in TMZ resistance, we investigated whether PLD1 affects the expression of TMZ resistance factors in GSCs. Interestingly, PLD1 depletion, but not TMZ treatment, markedly suppressed the expression of several TMZ resistance factors (*MGMT*, *ABCB1*, *ABCG2*, *PHF6*, *MMP16*, and *MCL1*), but not *HOXA9* and *HOXA10* [26, 27, 28, 29, 30], in non‐sorted GSC‐X01 (Figure 4A). TMZ treatment and PLD1 depletion further suppressed the expression of the TMZ resistance factors. IHC evaluation indicated that treatment of mice bearing intracranial GBM derived from PLD1‐depleted GSCs with TMZ markedly decreased the expression of TMZ resistance factors (other than *HOXA10*) in intracranial GBM tumors, compared with that when only TMZ treatment was performed (supplementary material, Figure S5). The Wnt/β‐catenin signaling pathway reportedly promotes stem cell properties and resistance to radio/chemotherapy in GBM [31, 32]. In the present study, the depletion of β‐catenin had no effect on the expression of the TMZ resistance factors (supplementary material, Figure S6A). Recent evidence indicates that dysregulation of miRNAs (miRs) is important in the development and progression of numerous cancers, including GBM [33]. Our previous miRNA array analysis of PLD1 inhibitor‐treated colorectal cancer (CRC) cells identified miRs induced by PLD1 inhibitor [4]. Based on the bioinformatics approach, we found that the 3′‐untranslated region (UTR) of the TMZ resistance factors contained the putative binding site(s) of miR‐320a and miR‐4496 upregulated by PLD1 inhibition (supplementary material, Figure S6B). The 3′‐UTRs of *ABCB1*, *ABCG2*, and *MGMT* contain putative miR‐4496 target site(s), and the 3′‐UTRs of *PHF6*, *MMP16*, and *MCL1* contain putative binding sites of both miR‐320a and miR‐4496 (supplementary material, Figure S6B). Interestingly, pre‐miR‐320a and pre‐miR‐4496 significantly suppressed expression of the TMZ resistance factors, except for *HOXA10*, which does not contain putative miR target sites (supplementary material, Figure S6C). Moreover, TMZ had no effect on the luciferase activity of the 3′‐UTRs of TMZ resistance genes (Figure 4B). The combination of PLD1 depletion and TMZ significantly decreased the luciferase activity of the 3′‐UTRs of *ABCB1*, *ABCG2*, and *MGMT*. The activity was recovered by deletion of the miR‐4496 binding site(s), whereas anti‐miR‐4496 did not reduce the luciferase activity of the 3′‐UTR of the TMZ resistance factors (Figure 4B). Furthermore, the combination of PLD1 depletion and TMZ significantly decreased the luciferase activity of the 3′‐UTRs of *PHF6*, *MMP16*, and *MCL1*. Activity was recovered by deletion of both the miR‐320a and the miR‐4496 binding sites. However, anti‐miR‐320a/‐4496 did not reduce the luciferase activity of the 3′‐UTRs of these TMZ resistance genes (Figure 4B). These findings suggest that the combination of PLD1 depletion and TMZ downregulates the levels of TMZ resistance factors via miR‐320a and miR‐4496. However, the combined use of TMZ and the miRs did not further promote apoptosis, compared with TMZ alone. In addition, pre‐miR‐320a/‐4496 greatly reduced the protein levels of the TMZ resistance factors (except for *HOXA10*) in CD44^High^ GSCs (Figure 4D). Moreover, PLD1 expression was inversely correlated with the levels of miR‐320a and miR‐4496 in CD44^High^ GSCs and U251‐TMZ‐R, but not in CD44^Low^ GSCs and wild‐type U251‐MG cells (Figure 4E). PLD1 expression significantly correlated with the levels of TMZ resistance factors (except for *HOXA9* and *HOXA10*) as assessed in the TCGA GBM expression profile database [34] (supplementary material, Figure S7). Collectively, these results suggest that PLD1 depletion sensitizes resistance to TMZ in GSCs via miR‐320a/4496‐mediated downregulation of TMZ resistance factors.

### Treatment of PLD1‐depleted GSCs with TMZ upregulates expression of miR‐320a and miR‐4496 via E2F1 and Sp1

**Figure 5:**
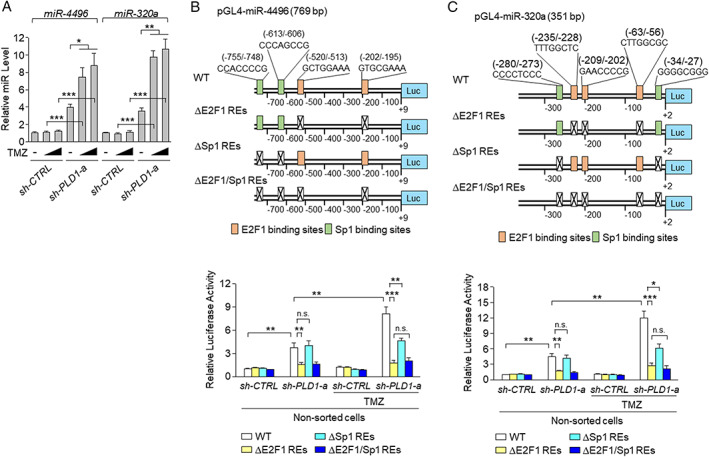
Combination of TMZ with PLD1 depletion in GSCs upregulates the expression of miR‐320a and miR**‐**4496 via E2F1 and Sp1. (A) RT‐qPCR analysis of miR‐320a and miR‐4496 in GSC_X01 transduced with sh‐CTRL or sh‐PLD1‐a and either untreated or treated with TMZ (50 and 100 μm). (B) Schematic representation for the promoter luciferase constructs of miR‐4496 containing wild‐type (WT) and mutant binding sites of E2F1 and Sp1. The promoter assays of miR‐4496 were performed under the indicated conditions. (C) Schematic representation for the promoter luciferase constructs of miR‐320a containing WT and mutant binding sites of E2F1 and Sp1. The promoter assays of miR‐320a were performed under the indicated conditions. Results are representative of at least three independent experiments and are presented as the mean ± SEM (A–C). *p < 0.05; **p < 0.01; ***p < 0.001, by Student's *t*‐test; n.s., not significant.

We investigated whether TMZ and/or PLD1 depletion could affect the expression of miR‐320a and miR‐4496. PLD1 depletion, but not TMZ treatment, significantly increased the expression levels of the miRs, which were further enhanced by the combination of PLD1 depletion and TMZ, relative to PLD1 depletion alone (Figure 5A). Targeting of PLD1 induces E2F1‐mediated miR‐4496 expression [4]. E2F1 and Sp1 reportedly superactivate their transcription via functional interaction [35]. The role of the E2F1/Sp1 interaction seems to be promoter‐specific and results in either synergistic activation [36] or repression of transcription [37]. E2F1 and Sp1 binding sites were identified in the miR‐320a and miR‐4496 promoters (Figure 5B,C). Deletion of the E2F1 binding sites in the miR‐320a and miR‐4496 promoters significantly decreased the promoter activity of the miRs that was induced by PLD1 depletion. However, deletion of Sp1 binding sites did not affect the promoter activity caused by PLD1 depletion (Figure 5B,C). The combined use of PLD1 depletion and TMZ further increased the promoter activity of the miRs (Figure 5B,C). Depletion of Sp1 and/or E2F1 binding sites significantly decreased the promoter activity of the miRs that had been stimulated by the combination (Figure 5B,C). Whether the depletion of Sp1 response elements affected the promoter activity induced by PLD1 depletion was not determined. However, E2F1 and Sp1 might synergistically activate the transcription of miR‐320a and miR‐4496, probably via a functional interaction by the combinational treatment. Taken together, these results suggest that the combined treatment of TMZ and PLD1 depletion in GSCs enhances the expression of miR‐320a and miR‐4496 via E2F1 and Sp1.

### Pharmacological inhibition of PLD1 attenuates the tumor‐initiating capacity of GSCs and sensitizes GSC‐derived intracranial tumors to TMZ

**Figure 6:**
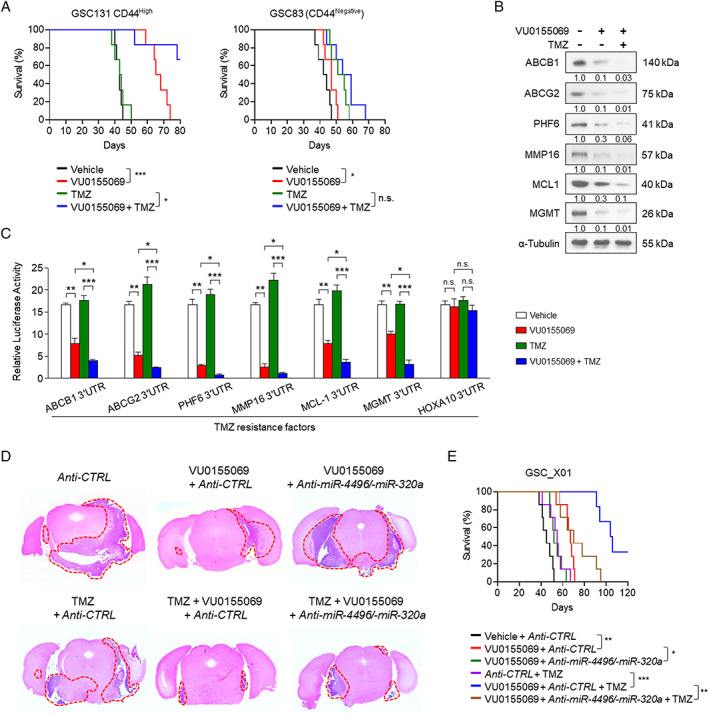
Pharmacological inhibition of PLD1 attenuates the tumor‐initiating capacity of GSCs and sensitizes GSC‐derived intracranial tumors to TMZ. (A) CD44^High^ population of GSC131 and CD44^Negative^ population of GSC‐83 were intracranially transplanted into the brains of immunocompromised NOD‐SCID mice (6 × 10^4^ cells per mouse), followed by intraperitoneal administration of VU0155069 (10 mg/kg) and/or TMZ (20 mg/kg) (*n* = 6 per group). Survival of mice (*n* = 6 per group) was done using the Kaplan–Meier model with two‐sided log‐rank test. *p < 0.05; ***p < 0.001. (B) After the CD44^High^ population of GSC_X01 was pretreated with VU0155069, the cells were treated with TMZ for 72 h, and the expression levels of the indicated proteins were analyzed by immunoblotting. (C) The luciferase activities of 3′‐UTR reporters of the indicated genes were measured in monolayers of GSCs treated with VU0155069 alone or in combination with TMZ (100 μm). (D) The CD44^High^ population of GSC_X01 was not transduced or was transduced with anti‐miR‐320a/miR‐4496 and intracranially transplanted into the brains of immunocompromised NOD‐SCID mice. The mice were treated with the indicated drugs (*n* = 7 per group). Representative images of hematoxylin and eosin‐stained sections of mice brains harvested on day 35 after transplantation from the indicated groups. The areas of red dotted lines show tumor sizes in the brains with the indicated groups. (E) Survival of mice was evaluated (*n* = 7 per group) using the Kaplan–Meier model with two‐sided log‐rank test. **p < 0.01; ***p < 0.001. The results are representative of at least three independent experiments and are presented as the mean ± SEM (B, E). *p < 0.05; **p < 0.01; ***p < 0.001, by Student's *t*‐test; n.s., not significant.

We assessed the effects of a PLD1 inhibitor on GSCs to evaluate the clinical implications. VU0155069 is a selective PLD1 inhibitor [38]. This inhibitor did not affect sphere formation in mNPCs (supplementary material, Figure S8A), whereas it significantly suppressed sphere formation in CD44^High^ GSCs derived from tumors transplanted with GL26 (supplementary material, Figure S8A). Moreover, the PLD1 inhibitor did not affect the apoptosis of mouse NPCs but enhanced the apoptosis of CD44^High^ mouse GSCs (supplementary material, Figure S8B). Co‐treatment involving the PLD1 inhibitor and TMZ further promoted the apoptosis of GSCs (supplementary material, Figure S8B). PLD1 inhibition, but not TMZ treatment, significantly improved the survival of mice intracranially implanted with CD44^High^ GSCs or GL26 (Figure 6A and supplementary material, Figure S8C). The combined treatment further increased the survival of mice, compared with the PLD1 inhibitor alone. TMZ or the PLD1 inhibitor also increased the survival of mice intracranially implanted with CD44^Negative^ GSC83, a PN subtype of GSCs, but their combined treatment showed no difference in the survival of the mice, compared with mice treated solely with TMZ (Figure 6A). Although the PLD1 inhibitor displayed greater therapeutic efficacy in mice implanted with a CD44^High^ population of GSCs, overall survival benefits were still observed in mice implanted with CD44^Negative^ PN‐GSCs (Figure 6A). Co‐treatment of the PLD1 inhibitor with TMZ in GSC‐X01 further reduced the expression of TMZ resistance factors relative to the PLD1 inhibitor alone (Figure 6B). Moreover, the PLD1 inhibitor markedly decreased the luciferase activity of 3′‐UTRs of the TMZ resistance genes (except for *HOXA10*), which was further reduced by combined treatment (Figure 6C). The PLD1 inhibitor alone greatly reduced GBM tumor formation and increased the survival of mice intracranially implanted with CD44^High^ GSCs (Figure 6D,E). Combinational therapy markedly abrogated intracranial tumors and significantly increased the survival of mice with GBM tumors, relative to PLD1 inhibition alone. Anti‐miR‐320a/‐4496 reversed the effects of PLD1 inhibition and combinational therapy (Figure 6D,E). Collectively, these results indicate that PLD1 inhibition sensitizes GSC‐derived intracranial tumors to TMZ via the miRs and supports the clinical application of this combinational therapy for treatment of GBM.

## Discussion

Our results demonstrated that PLD1‐based TMZ‐resistant mechanisms in GSCs represent crucial nodes for therapeutic intervention. Patients with recurrent GBM who develop resistance to TMZ have limited therapeutic options. GSCs are the most therapy‐resistant type of tumor cells in malignant gliomas [22]. Elucidating the molecular basis of TMZ resistance could contribute to the development of logically designed combination therapies to block resistance in TMZ chemotherapy [39]. CD44, a marker of GSCs, and epidermal growth factor receptor (EGFR) are overexpressed in GBM. Both have been implicated in GBM progression, survival, and metastasis [40, 41, 42]. Aurora‐A kinase reportedly induces chemoresistance by upregulating CD44 and maintaining tumor stemness in cancer cells [43]. Thus, the combination of conventional chemotherapeutic drugs with an anti‐EGFR antibody or Aurora kinase inhibitor might represent a promising therapeutic strategy for the management of chemoresistant GBM. However, co‐treatment of TMZ with the anti‐EGFR antibody afatinib reportedly had little effect on overall survival in patients with recurrent GBM [44]. Recently, it has been demonstrated that the combination of natural compounds, including boswellic acid and quercetin, with TMZ and/or afatinib has a synergistic chemo‐sensitizing effect in the treatment of GBM [45, 46].

Accumulating evidence indicates the critical role of PLD1 in tumorigenesis and the potential involvement of PLD1 in chemoresistance. We found that PLD1 expression was upregulated in recurrent GBM, CD44^High^ GSCs, and TMZ‐treated CD44^Low^ GSCs, and was correlated with poor prognosis in GBM. PLD1 is a potential prognostic factor in CRC, and PLD1 expression is associated with poor prognosis of CRC patients [4, 47]. TMZ resistance was observed in CD44^High^ GSCs with high levels of PLD1 expression. However, resistance to TMZ was not evident in CD44^Low^ GSCs. PLD1 regulates the self‐renewal ability of GSCs, and PLD1 expression is correlated with GSC stemness during differentiation of GSCs. PLD1 may exert a tumorigenic role by enriching the GSC pool. Thus, PLD1 might be a new target for GSC treatment. PLD1 suppression was very influential on the outcome of radiotherapy. In GBM tumors, the expression of PLD1 has been correlated with the levels of TMZ resistance factors, including MGMT. However, the molecular mechanisms of these factors in TMZ resistance in GBM remain to be clarified.

miRs have provided new potential treatments for highly resistant GBM [48, 49, 50]. We have identified miRs regulated by PLD1 inhibitors in CRC cells [4]. We also found that miR‐320a and miR‐4496 upregulated by PLD1 inhibition attenuated self‐renewal, tumor‐initiating capacity, and chemoresistance [4, 51]. Based on the bioinformatics approach, we determined that miR‐320a and miR‐4496 have putative target site(s) in the 3′‐UTRs of TMZ resistance genes. PLD1 inhibition reduces the levels of TMZ resistance factors via the upregulation of miR‐320a and miR‐4496 and sensitizes GSC and GSC‐derived intracranial tumors to TMZ. miR‐320a and miR‐4496 induced by PLD1 depletion downregulated the expression of *MCL1*, an anti‐apoptotic gene that is a key mediator of cell survival and drug resistance in GBM [52]. Thus, PLD1‐regulated MCL1 expression may explain the effect of PLD1 in the apoptosis of GBM cells. Although TMZ alone marginally affected GSC‐derived GBM tumorigenesis, PLD1 inhibition alone or in combination with TMZ attenuated GBM tumorigenicity through miR‐320a/‐4496. The PLD1–miR‐320a/‐4496 axis acts as a new target of TMZ resistance. Targeting this axis is a potential therapeutic strategy against GSC‐derived GBM tumorigenesis. PLD1 inhibition specifically targets GSCs, but not normal NPCs. Pharmacological and genetic inhibition of PLD1 has no obvious side effects [53]. This strategy may be particularly advantageous in clinical practice. Hyaluronic acid, a main component of the extracellular matrix, is a natural ligand to CD44 and has been used as a targeting moiety for CD44‐overexpressed cancers, facilitating preferential uptake and potent therapeutic efficacy [54]. The targeting of CD44 using hyaluronic acid nanohydrogel for the delivery of quercetin and TMZ in GBM improved the therapeutic efficacy of TMZ [55, 56]. Thus, nanomedicine targeting CD44 in GBM to deliver anti‐PLD1 drugs or natural anti‐cancer molecules in combination with TMZ could offer a new therapeutic opportunity for GBM target therapy. PLD1 inhibition could be feasible in a clinical setting in GBM patients. Until now, no potential side effects of PLD1 inhibitors like VU0155069 have been described after systemic administration. We also did not observe any side effects using VU0155069. Given that CSCs display intrinsic resistance to radiotherapy and chemotherapy, our results suggest the possibility of using anti‐GBM therapies to overcome GSC‐mediated therapeutic resistance based on the inhibition of PLD1.

## Author contributions statement

DWK and DSM conceived the project and designed the study. DWK and WCH performed most of the experiments under the supervision of DSM. YNN and KSP assisted with some of the *in vitro* and *in vivo* experiments. YNN and KYC provided reagents and conceptual advice. DWK and DSM wrote the manuscript. All the authors discussed the results and commented on the manuscript.

## Supporting information


**Supplementary materials and methods**

**Figure S1.** PLD1 is upregulated in human GBM tissues
**Figure S2.** PLD activity and tumorsphere forming capacity are remarkably increased in the CD44^High^ population of GSCs relative to the CD44^Low^ population of GSCs
**Figure S3.** Effect of PLD1 depletion and TMZ on the viability, sphere formation, and self‐renewal capacity of the CD44^High^ population of GSCs
**Figure S4.** Effect of PLD1 depletion on the expression of MAP2 and population of CD44 and MAP2 in the GSCs
**Figure S5.** Treatment of TMZ in intracranial tumor with PLD1‐depleted GSCs reduces the expression of TMZ resistance proteins
**Figure S6.** Effect of β‐catenin depletion and pre‐miR‐320a/‐4496 on the expression of TMZ resistance genes
**Figure S7.** Expression of PLD1 is correlated with levels of TMZ resistance genes
**Figure S8.** Effect of PLD1 inhibitor on sphere formation and apoptosis in NPCs and GSCs

**Table S1.** The sequences of qPCR primers and 3′‐UTR cloning primers used in the study

## Data Availability

The data that support the findings of this study are available from the corresponding author DSM upon reasonable request.
