# Supplementary material for: Phospholipase D1 inhibition sensitizes glioblastoma to temozolomide and suppresses its tumorigenicity
Source: J Pathol. 2020 Sep 10;252(3):304–16. doi: 10.1002/path.5519 (PMC7693208; doi:10.1002/path.5519)
Supplement: Supplementary file 1 — Supplementary materials and methods [file PATH-252-304-s001.docx]

**Phospholipase D1 inhibition sensitizes glioblastoma to temozolomide and suppresses its tumorigenicity**

DW Kang, WC Hwang *et al. J Pathol* DOI: 10.1002/path.5519

**Supplementary materials and methods**

Reference numbers refer to the main text list

**PLD activity assay**

Phosphatidic acid (PA) production was measured using the Total Phosphatidic Acid Fluorometric Assay kit (#700240; Cayman Chemical, Ann Arbor, MI, USA) for *in vitro* analysis, according to the manufacturer’s protocol.

**Reagents and radiation exposure**

TMZ (S1237) was purchased from Selleck Chemicals. VU0155069 (13206) was purchased from Cayman Chemical. Cells were irradiated at room temperature using gamma rays from a Cs-137 blood irradiator (Eckert & Ziegler, Berlin, Germany) at a dose rate of 6.0 Gy/min. Non-irradiated controls were handled identically to the irradiated cells, with the exception of radiation exposure. After irradiation, cultures were incubated at 37 °C in an atmosphere of 5% CO_2_.

**Preparation of plasmids, shRNAs, and miRNA**

The shRNA lentiviral constructs against PLD1 or β-catenin in the pLKO vector were purchased from Sigma-Aldrich (St Louis, MO, USA) [4]. Precursor (Pre)-miR-4496 or -miR-320a lentiviral constructs in the pLenti-III-miR-GFP were purchased from ABM. Anti-miR-320a (shRNA targeting miR-320a) and anti-miR-4496 (shRNA targeting miR-4496) were cloned into lentiviral pEZX-mcherry vector (Genecopoeia, Rockville, MD, USA).

**Viral production and infection**

The sh-CTRL, sh-PLD1-a, sh-PLD1-b, sh-β-catenin, GFP-PLD1, FLAG-PLD1, and pre- or anti-miRNA lentivirus were produced using ViraPower™ Lentiviral Packaging Mix (Thermo Fisher Scientific, Waltham, MA, USA; #K4975-00). In brief, 293FT (Thermo Fisher Scientific, #R70007) cells were seeded at a density of 10^5^ cells per 35-mm diameter dish in DMEM (Hyclone, #SH30243.01) supplemented with 10% fetal bovine serum (FBS; Hyclone, #SH30084.03). After 18 h, cells were transfected. In this procedure, 10 μl of Lipofectamine 2000 (Thermo Fisher Scientific, #12566014) was added to 100 μl of DMEM and incubated for 20 min. Viral vector (1 µg) along with 0.9 mg of the appropriate gag/pol expression vector and 0.1 mg of VSVG expression vector was then added to the DMEM/Lipofectamine 2000 mixture, incubated for 30 min, and added to 293FT cells. The cells were then incubated overnight. The next day, fresh medium was added to the transfected 293FT cells. Viral supernatant was harvested at 48 and 72 h post-transfection, filtered, and added to the recipient cell lines with 6 μg/ml Polybrene (Sigma-Aldrich, #107689) for 12 h infection. After a 24 h incubation in the presence of viral particles, the medium was changed and cells were cultured for an additional 24 h.

**Transient transfection and reporter gene assay**

Following the manufacturer’s instructions, luciferase reporter plasmids, expression plasmids, or miRNAs were transiently transfected into cells with Lipofectamine 2000 and polyethyleneimine (#408727, Sigma Aldrich) reagents. Relative luciferase activity was obtained by normalization of firefly and *Renilla* luciferase activity. Dual-luciferase assay kits (E1910) were purchased from Promega (Madison, WI, USA).

**Reverse transcription-quantitative PCR (RT-qPCR)**

Total RNA was extracted using TRIzol reagent (#15596018, Thermo Fisher Scientific). RNA (3 μg) was reverse-transcribed to cDNA using the High Capacity cDNA Reverse Transcription Kit (#4368814, Thermo Fisher Scientific) according to the manufacturer’s instructions. Real-time qPCR was performed in triplicate. The final results were determined using a relative standard curve. Primer sets listed in supplementary material, Table S1 were used in qPCR to measure gene expression relative to *ACTB* or *18S*.

**Quantification of mature miRNA**

The TaqMan MicroRNA Reverse Transcription Kit (#4366596, Thermo Fisher Scientific) was used in combination with the miR-4496- or miR-320a-specific reverse transcription primers, followed by PCR using the indicated TaqMan specific primers and TaqMan™ Fast Universal PCR Master Mix (2×), no AmpErase™ UNG (#4352042, Thermo Fisher Scientific). *RNU6B* (#4427975, Thermo Fisher Scientific) was used as the normalization control.

**3'-UTR reporter constructs**

For construction of the human 3'-UTR reporter plasmids (relative to translation last nucleotide), the following domains were amplified from cDNA synthesized from the human normal colon RNA (#AM7986, Thermo Fisher Scientific): *ABCB1* (+1 to +380), *ABCG2* (+1 to +865), *PHF6* (+1 to +3118), *MMP16* (+1 to +1800), *MCL-1* (+1 to +2773), *MGMT* (+1 to +619), and *HOXA10* (+1 to +1296), and cloned into the XhoI or NotI site downstream of the *Renilla* luciferase gene in the vector psiCHECK-2 (#C8021, Promega). The indicated miRNA seed sequence binding sites in the 3'-UTRs of their targets were deleted completely (miR-4496 or/and miR-320a) using the QuikChange II Site-Directed Mutagenesis Kit (#200524; Agilent Technologies, Santa Clara, CA, USA). Primer sets listed in supplementary material, Table S1 were used in 3'-UTR cloning of the indicated genes.

**Cell viability assessment using trypan blue exclusion**

After a 72 h culture, cells were harvested, dissociated by trypsinization, and mixed 1:1 with trypan blue for 2 min. Viable (white) and dead (blue) cells were immediately counted using a hemocytometer.

**Differentiation assay**

GSCs were cultured in six-well plates or 60 mm dishes, and differentiation was induced using neurobasal medium lacking epidermal growth factor and basic fibroblast growth factors or supplemented with serum containing 10% FBS. At the indicated times, cells were harvested for immunoblotting, RT-qPCR, or flow cytometry analysis, as described above.

***In vitro* limiting dilution assay (LDA)**

To determine the number of sphere-forming units (SFUs), cells were cultured in serum-free medium with EGF (#cyt-217; PROSPEC, Israel) and bFGF (#cyt-218, PROSPEC). Defined numbers of cells were plated in each well in a 96-well plate. The highest and lowest cell numbers in a fixed volume of 200 μl per well were 100 000 and 10, respectively. At least six wells were seeded per cell concentration. Each sample and each condition were tested by plating cells in this dilution series, down to one cell per well. At the end of the experiment, we scored a binary output, with wells read as positive or negative. A positive well was defined as having at least one sphere and the negative wells had no spheres, based on visual inspection. The ELDA website (http://bioinf.wehi.edu.au/software/elda/index.html) was employed to calculate a sphere initiating cell frequency. We did not attempt to count multiple spheres in a well due to the inherent inaccuracy, since aggregation of spheres made it impossible to establish the sphere as clonal as in bulk culture. Sphere replating frequency was obtained from repeating the *in vitro* LDA with ten individual spheres. The average number of SFUs counted upon replating of ten LDAs derived from single spheres constituted the *in vitro* self-renewal assay.

**Immunoblotting**

Cell lysates were analyzed by immunoblotting assays, as described previously [5]. Enhanced chemiluminescence was used to detect the signal. The following antibodies were used: anti-GFAP (sc-56395, 1:1000), anti-MAP2 (sc-74421, 1:1000), anti-CD44 (sc-7297, 1:1000), anti-GFP (sc-9996, 1:1000), anti-mouse IgG (sc-2005, 1:2000), and anti-rabbit IgG (sc-2004, 1:2000) from Santa Cruz Biotechnology (Dallas, TX, USA); anti-ABCB1 (#MBS855876, 1:1000; MyBioSource, San Diego, CA, USA); anti-ABCG2 (ab63907, 1 μg/ml) and anti-MMP16 (ab73877, 1 μg/ml) from Abcam (Cambridge, UK); anti-PHF6 (NB100-68260, 1:1000) and anti-MCL-1 (NB100-56146, 1:1000) from Novus Biologicals (Centennial, CO, USA); anti-HOXA10 (GTX37412, 1:1000) from GeneTex (Irvine, CA, USA); anti-β-catenin (#610153, 1:2000) from BD Biosciences (San José, CA, USA); anti-MGMT (MS-470, 1:1000) from Thermo Fisher Scientific; anti-SOX2 (S9072, 1:1000) and anti-α-tubulin (T6074, 1:20 000) from Sigma-Aldrich; and active caspase-3 (#9661, 1:1000) from Cell Signaling Technology (Danvers, MA, USA). The signal densities on the blots were measured with ImageJ software (Rasband, WS, ImageJ, U.S. National Institutes of Health, Bethesda, Maryland, USA; https://imagej.nih.gov/ij/, 1997–2018) and normalized using anti-α-tubulin antibody.

**Immunohistochemistry (IHC)**

Anti-Ki67 (ab15580, 1:300, Abcam), anti-ABCG2 (ab24115, 1:300, Abcam), anti-MMP16 (2 μg/ml, Abcam), anti-HOXA10 (ab191470, 1:300, Abcam), anti-ABCB1 (HPA002199, 1:200, Sigma-Aldrich), anti-active caspase-3 (1:300), anti-MCL-1 (39224S, 1:300, Cell Signaling Technology), and anti-PHF6 (NB100-68261, 1:200, Novus Biologicals) antibodies were used as the primary antibodies. IHC analysis of diaminobenzidine (DAB) staining was performed using an UltraTek HRP Anti-Polyvalent Lab Pack (#UHP125; ScyTek, Logan, UT, USA). DAB-stained specimens were observed using a general optical microscope with an AxioCam ICc5 camera (Carl Zeiss, Oberkochen, Germany). Hematoxylin and eosin staining of the entire brain was conducted using a Zeiss MIRAX (Carl Zeiss). Images were processed with equivalent parameters using the ZEN Light Edition software (Carl Zeiss). High-grade glioma tissue microarray slides (A221 I, IV, V) were purchased from ISU Abxis Co Ltd (Republic of Korea). Each array included 109 cases of normal (*n* = 6), anaplastic astrocytoma III (*n* = 12), and glioblastoma IV (*n* = 91) primary tissues.

**Expression data from the TCGA glioblastoma multiforme dataset**

TCGA gene RNA sequencing data of tumors in cohorts based on the TCGA GBM expression profile database (Cancer Genome Atlas Research Network, 2008) were downloaded from the TCGA website (<https://tcga-data.nci.nih.gov/tcga/tcgaDownload.jsp>).
