# Supplementary material for: Phospholipase D1 inhibition sensitizes glioblastoma to temozolomide and suppresses its tumorigenicity
Source: J Pathol. 2020 Sep 10;252(3):304–16. doi: 10.1002/path.5519 (PMC7693208; doi:10.1002/path.5519)
Supplement: Supplementary file 2 — Figure S1. PLD1 is upregulated in human GBM tissues Figure S2. PLD activity and tumorsphere forming capacity are remarkably increased in the CD44High population of GSCs relative to the CD44Low population of GSCs Figure S3. Effect of PLD1 depletion and TMZ on the viability, sphere formation, and self‐renewal capacity of the CD44High population of GSCs Figure S4. Effect of PLD1 depletion on the expression of MAP2 and population of CD44 and MAP2 in the GSCs Figure S5. Treatment of TMZ in intracranial tumor with PLD1‐depleted GSCs reduces the expression of TMZ resistance proteins Figure S6. Effect of β‐catenin depletion and pre‐miR‐320a/‐4496 on the expression of TMZ resistance genes Figure S7. Expression of PLD1 is correlated with levels of TMZ resistance genes Figure S8. Effect of PLD1 inhibitor on sphere formation and apoptosis in NPCs and GSCs [file PATH-252-304-s002.docx]

**Phospholipase D1 inhibition sensitizes glioblastoma to temozolomide and suppresses its tumorigenicity**

DW Kang, WC Hwang *et al. J Pathol* DOI: 10.1002/path.5519

**Supplementary Figures S1–S8**


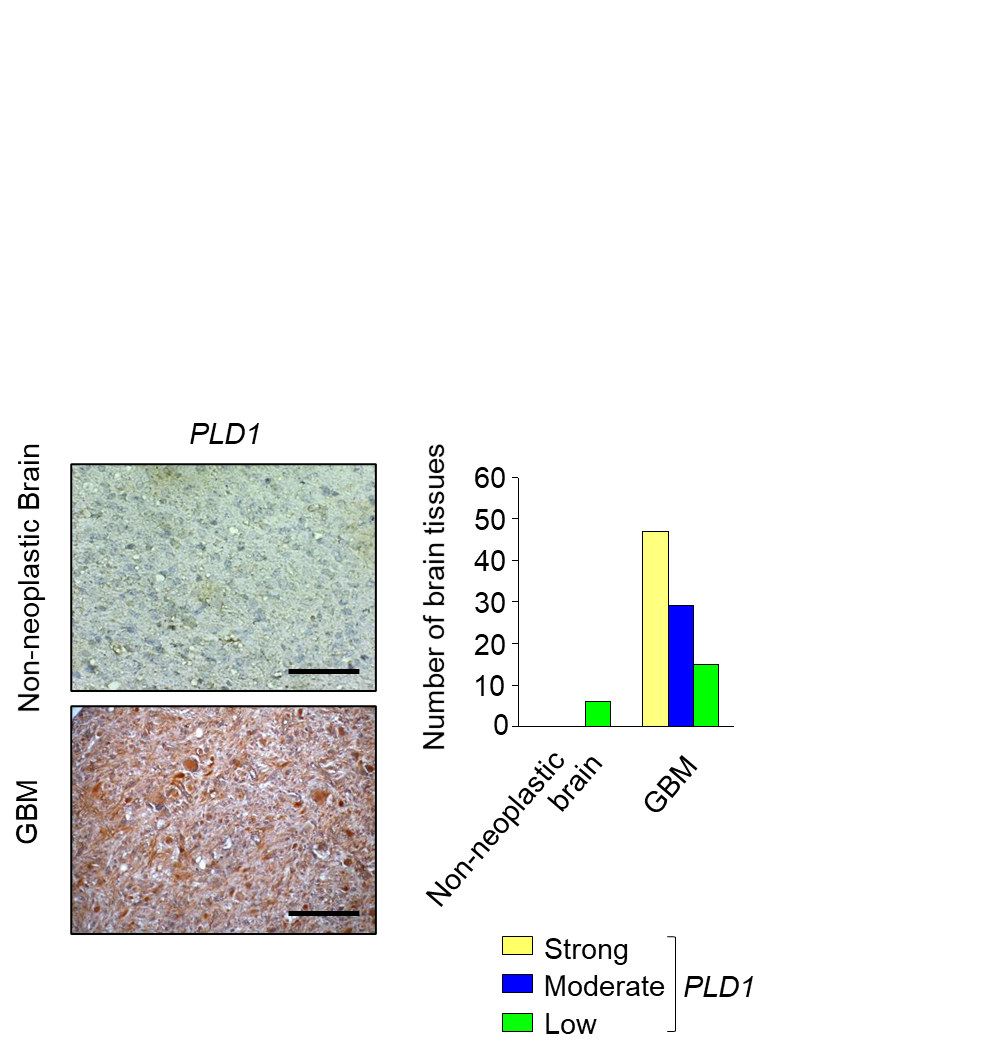


**Figure S1.** PLD1 is upregulated in human GBM tissues. Representative immunohistochemical images of non-neoplastic brain and GBM tissues in TMA (left). Bar = 50 μm (left). Graphs summarizing chi-squared analysis of IHC (right).

**
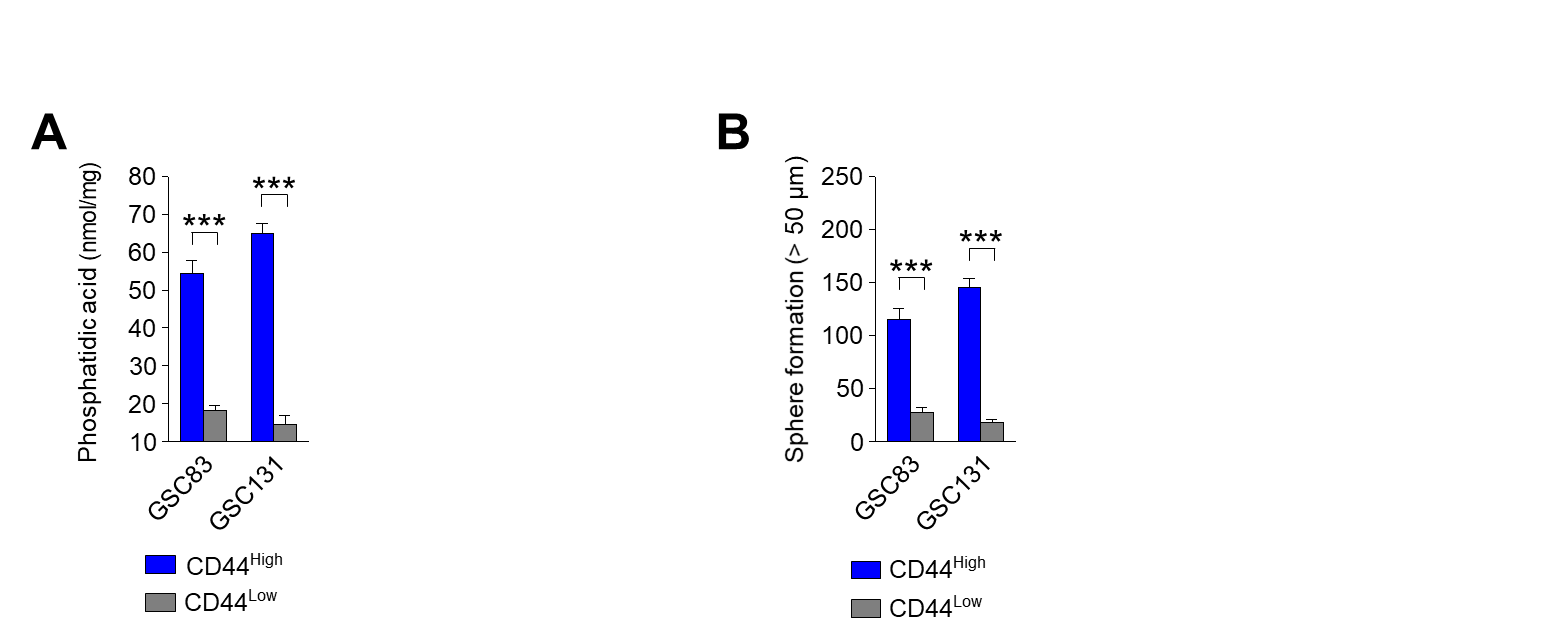
**

**Figure S2.** PLD activity and tumorsphere forming capacity are remarkably increased in the CD44^High^ population of GSCs relative to the CD44^Low^ population of GSCs. (A) Levels of cellular PA in the CD44^High^ and CD44^Low^ population sorted by MACS and FACS from MES GSCs. (B) Quantification of tumorsphere formation in the indicated populations sorted by MACS and FACS from MES GSCs. Data represent the mean ± SEM of three independent experiments. ****p* < 0.001, by Student’s *t*-test.

**
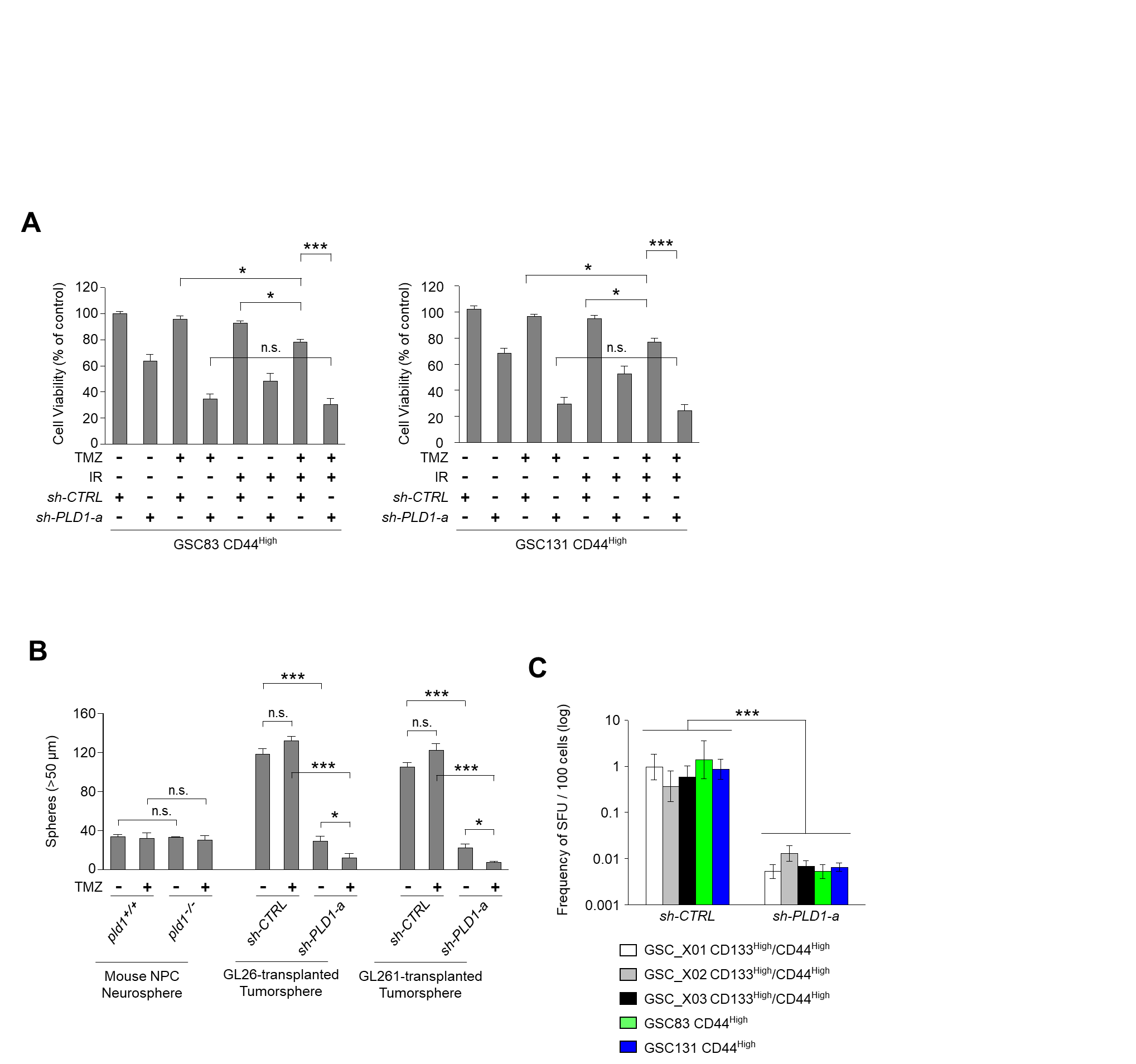
**

**Figure S3.** Effect of PLD1 depletion, TMZ or IR on the viability, sphere formation, and self-renewal capacity of the CD44^High^ population of GSCs. (A) Effect of TMZ and/or IR on the viability of the indicated CD44^High^ GSCs transduced with sh-PLD1-a or sh-CTRL. The viability was measured by trypan blue exclusion assay. The number of tumorspheres was measured at 72 h. (B) The number of neurospheres and tumorspheres was determined in NPCs from *pld1*^+/+^ and *pld1*^−/−^ mice, GL26-transplanted CD44^High^ GSCs, and GL261-transplanted CD44^High^ GSCs, under the indicated conditions. (C) *In vitro* LDA of sphere-forming units by PLD1 depletion in the indicated population of GSCs; error bars represent 95% CI (confidence intervals). Data represent the mean ± SEM of three independent experiments. **p* < 0.05; ****p* < 0.001, by Student’s *t*-test. n.s., not significant.

**
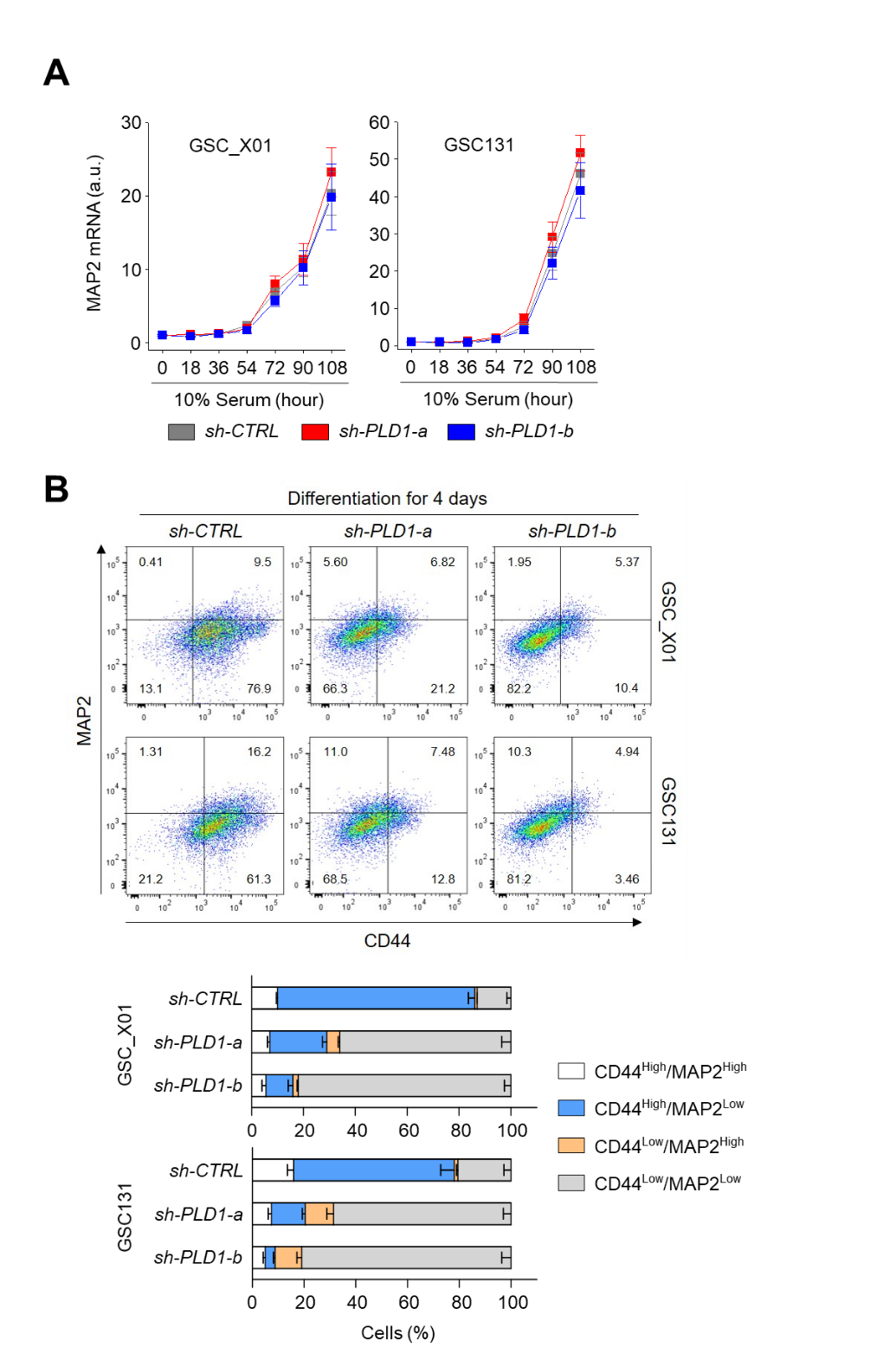
**

**Figure S4****.** Effect of PLD1 depletion on the expression of MAP2 and population of CD44 and MAP2 in the GSCs. (A) Effects of PLD1 depletion on the expression of MAP2 during differentiation of the GSCs for the indicated time, as analyzed by RT-qPCR. (B) Effects of PLD1 depletion on the population of CD44 and MAP2 during differentiation of the indicated GSCs. Data represent the mean ± SEM of three independent experiments.

**
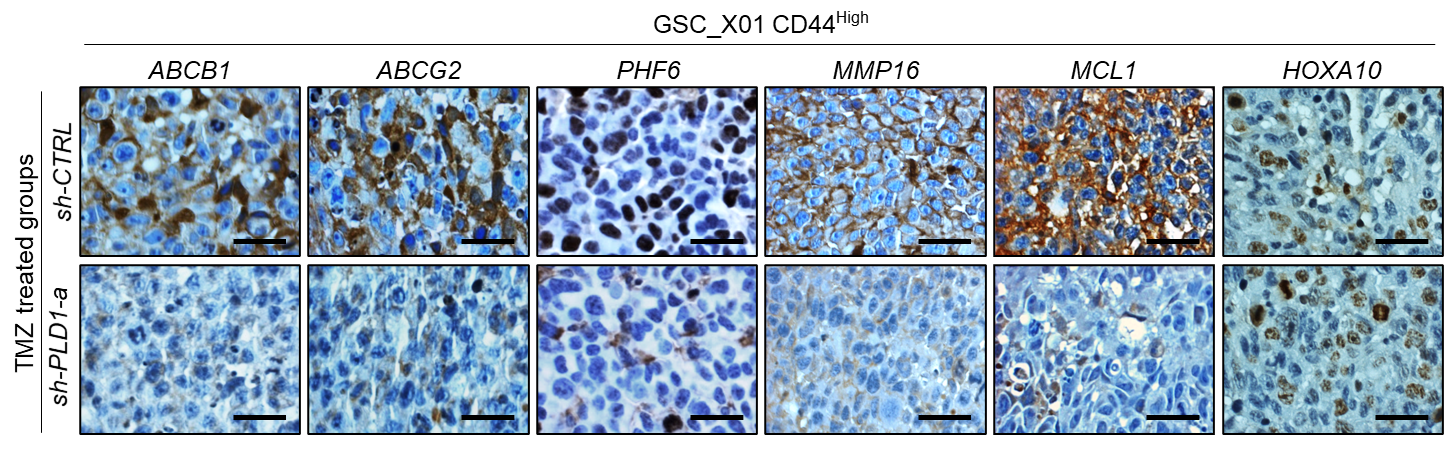
**

**Figure S5.** Treatment of TMZ in intracranial tumor with PLD1-depleted GSCs reduces the expression of TMZ resistance proteins. Expression of the indicated proteins in tumors of mice bearing PLD1-depleted GSC_X01 CD44^High^ cells was analyzed by IHC. Representative images were selected from at least three different fields.

**
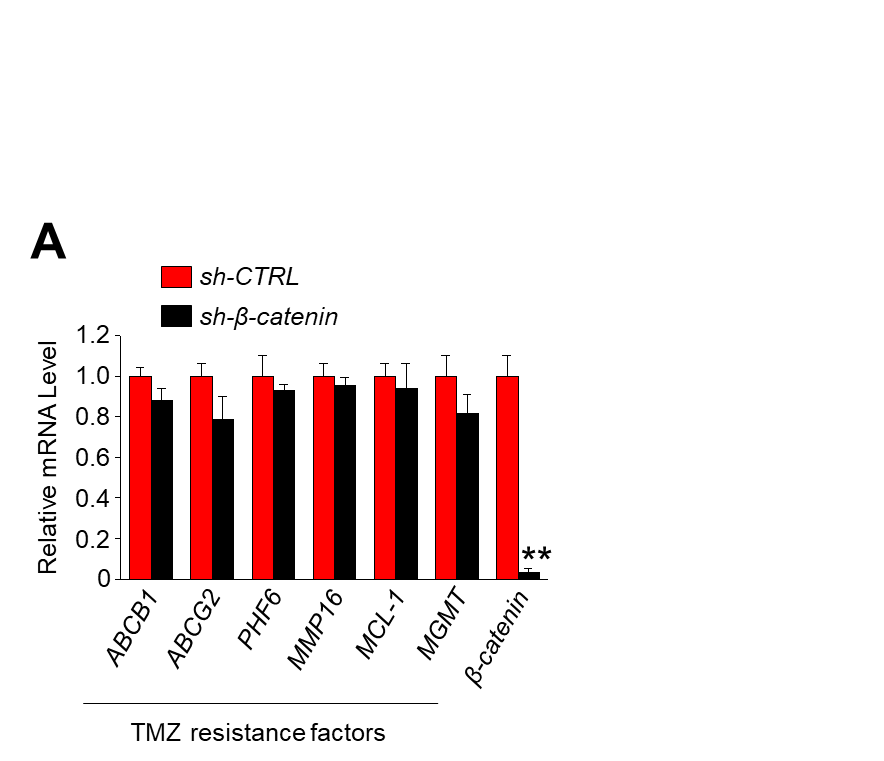
**

**
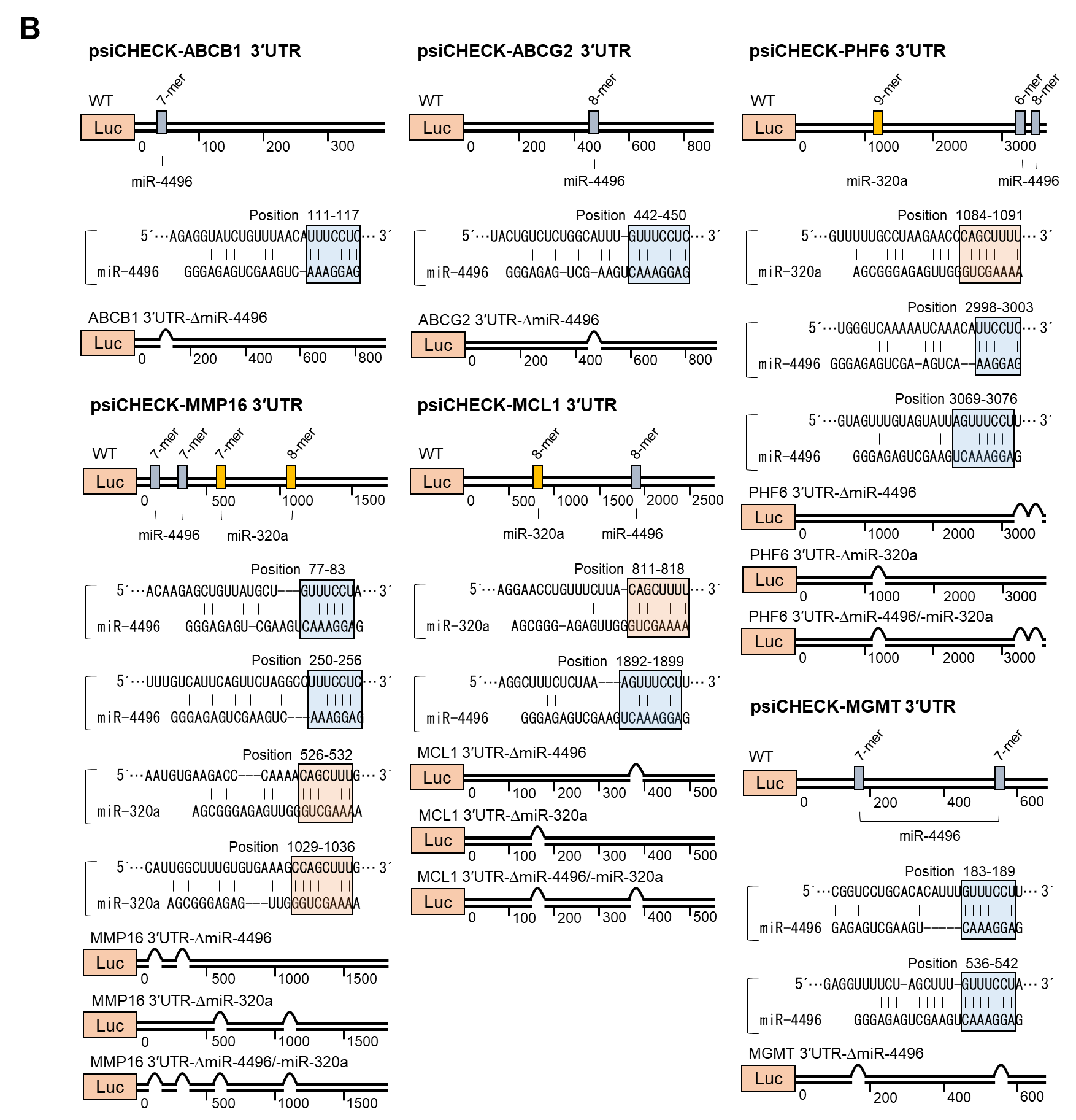
**

**
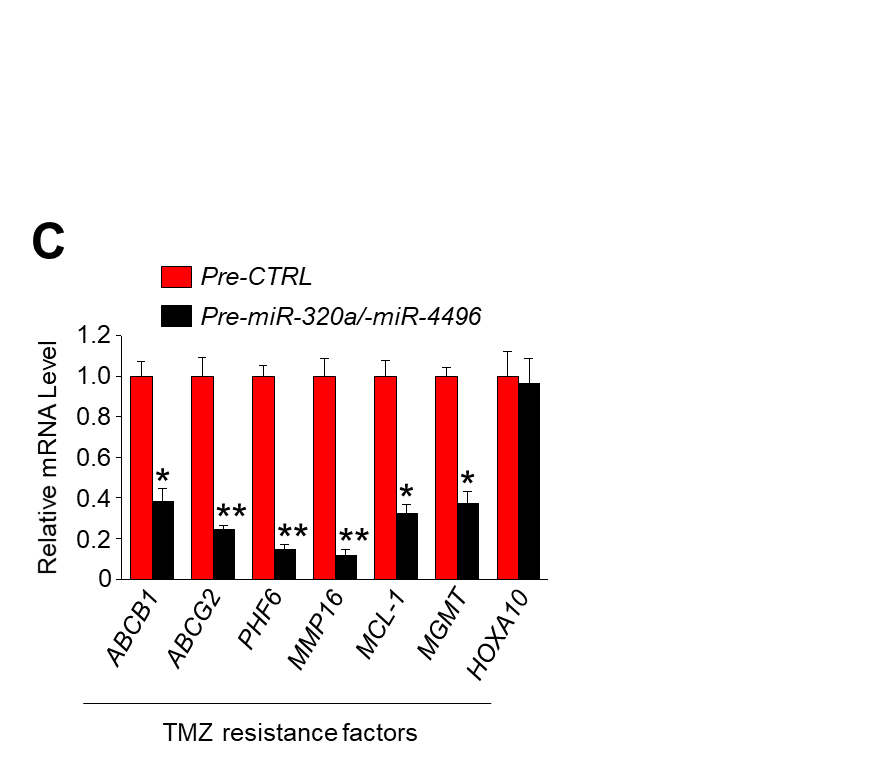
**

**Figure S6.** Effect of β-catenin depletion and pre-miR-320a/-4496 on the expression of TMZ resistance genes. (A) Effect of β-catenin depletion on the expression of TMZ resistance genes in GSC_X01, as analyzed by q-PCR. (B) Schematic representation of the 3'-UTR luciferase constructs of TMZ resistance factors. ∆miR-4496 was generated by deletion of miRNA binding sites in the 3'-UTR luciferase constructs of ABCB1, ABCG2, and MGMT. ∆miR-4496 or/and ∆miR-320a were generated by deletion of miRNA binding sites in the 3'-UTR luciferase constructs of PHF6, MMP16, and MCL1. (C) Effect of pre-miR-320a/-4496 depletion on the expression of TMZ resistance genes in GSC_X01, as analyzed by q-PCR. Data represent the mean ± SEM of three independent experiments. **p* < 0.05; ***p* < 0.01, by Student’s *t*-test. n.s., not significant.

**
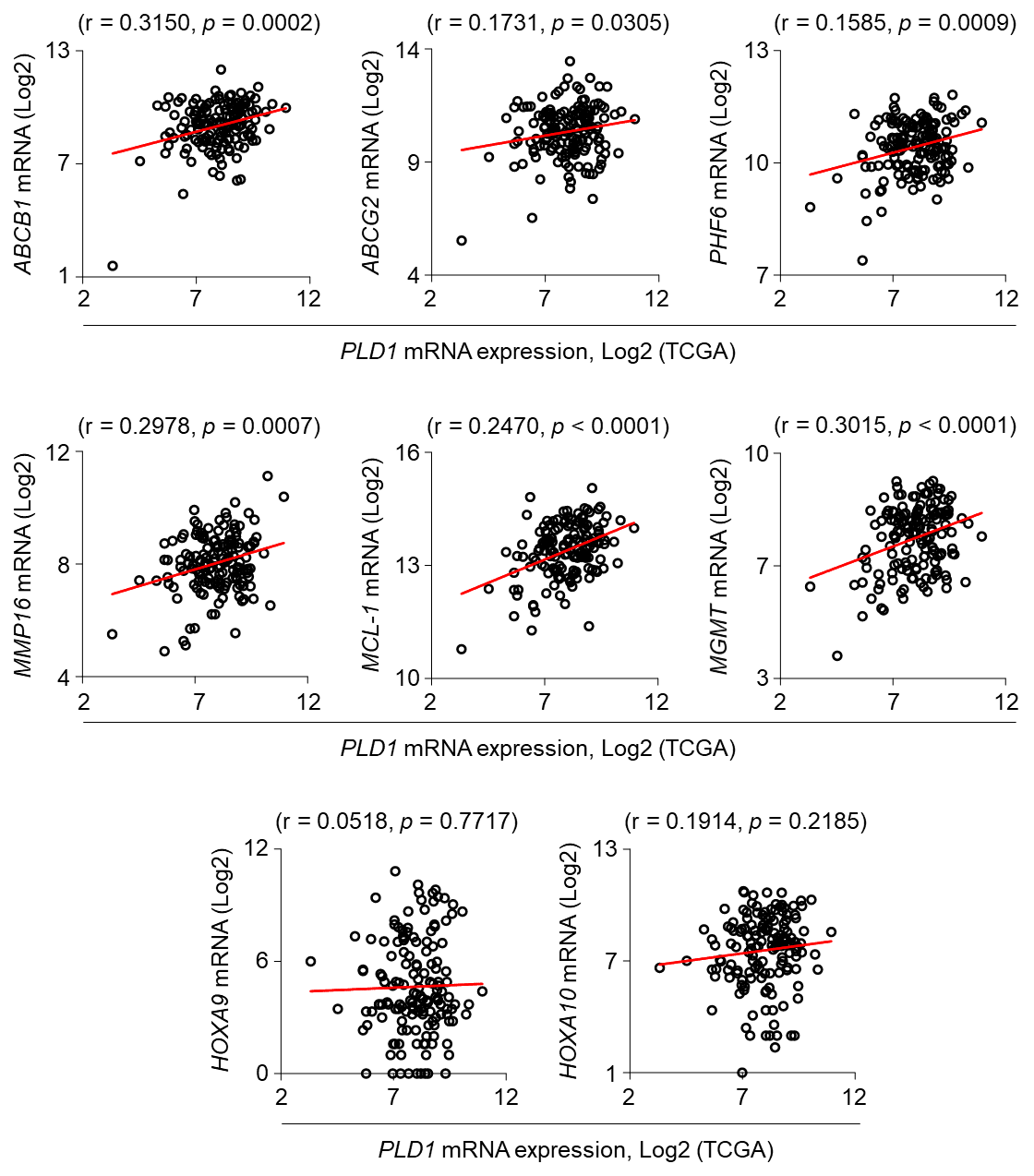
**

**Figure S7.** Expression of PLD1 is correlated with levels of TMZ resistance genes. The correlation of expression of the indicated genes with *PLD1* mRNA levels was examined in the TCGA GBM database. Spearman’s correlation coefficient (r) is provided along with its statistical significance. The red lines represent the best-fit curves.

**
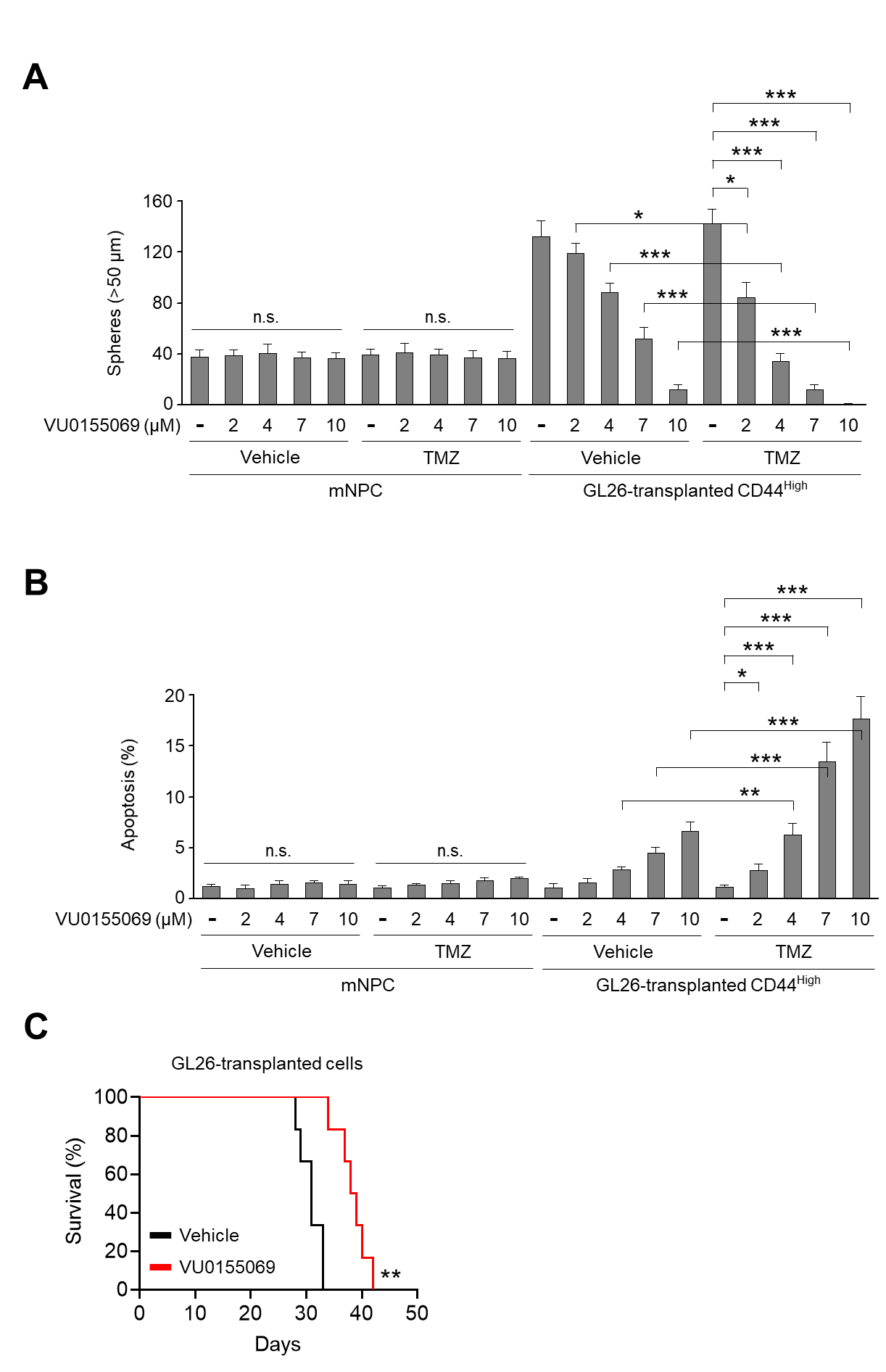
**

**Figure S8.** Effect of PLD1 inhibitor on the sphere formation and apoptosis in NPCs and GSCs. After mNPCs and GL26-transplanted CD44^High^ cells were pretreated with VU0155069 in a dose-dependent manner under sphere culture conditions, the cells were treated with TMZ for 96 h. The number of spheres (A) and apoptosis (B) were measured 5 days after initial culture. A minimum cut-off of 50 μm diameter was used. (C) GL26 cells were intracranially transplanted into the brains of immunocompromised NOD-SCID mice (6 × 10^4^ cells per mouse), followed by intraperitoneal administration of VU0155069 (10 mg/kg) (*n* = 8 per group) and survival of mice (Kaplan–Meier model with two-sided log-rank test) was evaluated. Results are representative of at least three independent experiments and are shown as the mean ± SEM. **p* < 0.05; ***p*< 0.01; ****p*< 0.001, by Student’s *t*-test. n.s., not significant.
