# Supplementary material for: Phospholipase D1 inhibition sensitizes glioblastoma to temozolomide and suppresses its tumorigenicity
Source: J Pathol. 2020 Sep 10;252(3):304–16. doi: 10.1002/path.5519 (PMC7693208; doi:10.1002/path.5519)
Supplement: Supplementary file 3 — Table S1. The sequences of qPCR primers and 3′‐UTR cloning primers used in the study [file PATH-252-304-s003.docx]

**Phospholipase D1 inhibition sensitizes glioblastoma to temozolomide and suppresses its tumorigenicity**

DW Kang, WC Hwang *et al. J Pathol* DOI: 10.1002/path.5519

**Table S1.** The sequences of qPCR primers and 3'-UTR cloning primers used in the study

| **Gene symbol** | **Forward primer (5' to 3')** | **Reverse primer (5' to 3')** |
| --- | --- | --- |
| *PLD1* | AATCGTTGGAGGTTGGACTG | AGCATTTCGAGCTGCTGTTGAA |
| *PLD2* | CATCCAGGCCATTCTGCAC | GTGCTTCCGCAGACTCAAGG |
| *CTNNB1* | GGTTGCCTTGCTCAACAAAA | TCCCAAGGAGACCTTCCATC |
| *CD133* | CAAAGAGGCGTTGGAGAAC | GCAGGCTAGTTTTCACGCTG |
| *CD44* | TCCAACACCTCCCAGTATGACA | GGCAGGTCTGTGACTGATGTACA |
| *BMI1* | CCAGGGCTTTTCAAAAATGA | GCATCACAGTCATTGCTGCT |
| *ID3* | CTGGACGACATGAACCACTG | GTAGTCGATGACGCGCTGTA |
| *ALDH1A1* | TCGTCTGCTGCTGGCGACAA | AGCCCAACCTGCACAGTAGCG |
| *ABCB1* | GCCTGGCAGCTGGAAGACAAATAC | ATGGCCAAAATCACAAGGGTTAGC |
| *ABCG2* | CCGCGACAGCTTCCAATGACCT | GCCGAAGAGCTGCTGAGAACTGTA |
| *PHF6* | AGCGCAAATGTGGCTTTTGT | GAGCATGCACTTATGGTGCG |
| *MMP16* | AGCACTGGAAGACGGTTGG | CTCCGTTCCGCAGACTGTA |
| *MGMT* | ACCGTTTGCGACTTGGTACT | CGGGGAACTCTTCGATAGCC |
| *MCL-1* | CCAAGGCATGCTTCGGAAA | TCACAATCCTGCCCCAGTTT |
| *EZH2* | GCGGATAAAGACCCCACCAA | GTATCCACATCCTCAGCGGG |
| *FOXM1* | GGAGGAAATGCCACACTTAGCG | TAGGACTTCTTGGGTCTTGGGGTG |
| *HOXA9* | ccacgcttgacactcacact | agttggctgctgggttattg |
| *HOXA10* | AGTACCCGGCTACTTCCGCCTGTC | CGCTCTTTGCTGTGAGCCAGTTG |
| *GFAP* | TGGTGAAGACCGTGGAGATGC | TCCTCCCCTTCTCTCCTTCCT |
| *MAP2* | CTGCTTTACAGGGTAGCACAA | TTGAGTATGGCAAACGGTCTG |
| *ACTB* | AGAAAATCTGGCACCACACC | AGAGGCGTACAGGGATAGCA |
| *18S* | CGTCTGCCCTATCAACTTTCG | TTCCTTGGATGTGGTAGCCG |
| **3'-UTR cloning** | **Sense (5' to 3')** | **Antisense (5' to 3')** |
| *CTNNB1* | AAACTCGAG CAATCAGCTGGCCTGGTTTGATAC | GCGGCCGCC AATAGTTTTTGATCAAAAACATGAAATAGATC |
| *ABCB1* | AAACTCGAG actctgactgtatgagatgttaaatacttt | GCGGCCGCC tcacatgaaagtttagttttattatag |
| *ABCG2* | AAACTCGAG atttccccttaattcagtatgatttatcct | GCGGCCGCC atccaggagtggtcagattcctttatgaag |
| *PHF6* | AAACTCGAG gttcatgggacagagttagaaaactgg | GCGGCCGCC gtcatttattgaatgaatagtatatgcaag |
| *MMP16* | AAACTCGAG tgtagggttttttcttctttctttcttttgcag | GCGGCCGCC ctggctttcacacaaagccaatgtctg |
| *MCL1* | AAACTCGAG ccttactgtaagtgcaatagttgac | GCGGCCGCC GGAaacacactacatttgacaaccaac |
| *MGMT* | AAACTCGAG gtatgtgcagtaggatggatgtttgag | GCGGCCGCC cttggcaaatggttattttctattcacct |
| *HOXA10* | AAACTCGAG tgaatctccaggcgacgcggttttttcac | GCGGCCGCC gcttcattccacagcttttattctataag |
